# Physical multimorbidity and dynapenic abdominal obesity among older adults from low- and middle-income countries

**DOI:** 10.1038/s43856-025-01037-9

**Published:** 2025-07-29

**Authors:** Lee Smith, Guillermo F. López Sánchez, Nicola Veronese, Pinar Soysal, Mark A. Tully, Karel Kostev, Laurie Butler, Helen Keyes, Yvonne Barnett, Jae Il Shin, Ai Koyanagi

**Affiliations:** 1 Centre for Health Performance and Wellbeing, Anglia Ruskin University, Cambridge, UK; 2 Department of Public Health, Faculty of Medicine, Biruni University, Istanbul, Turkey; 3 Division of Preventive Medicine and Public Health, Department of Public Health Sciences, School of Medicine, University of Murcia, Murcia, Spain; 4 Faculty of Medicine, Saint Camillus University, Rome, Italy; 5 Primary Care Department, Azienda ULSS (Unità Locale Socio Sanitaria) 3 “Serenissima”, 30174 Venice, Italy; 6 Department of Geriatric Medicine, Faculty of Medicine, Bezmialem Vakif University, Istanbul, Turkey; 7 School of Medicine, Ulster University, Londonderry, Northern Ireland UK; 8 University Clinic of Marburg, Marburg, Germany; 9 School of Psychology and Sport Science, Anglia Ruskin University, Cambridge, UK; 10 Department of Pediatrics, Yonsei University College of Medicine, Seoul, Republic of Korea; 11 Severance Underwood Meta-Research Center, Institute of Convergence Science, Yonsei University, Seoul, Republic of Korea; 12 Research and Development Unit, Parc Sanitari Sant Joan de Déu, Dr. Antoni Pujadas, Sant Boi de Llobregat, Barcelona Spain

**Keywords:** Diseases, Health care

## Abstract

**Background:**

As far as we are aware, to date, there are no studies on the association between dynapenic abdominal obesity (DAO) and physical multimorbidity (i.e., ≥2 chronic conditions). Thus, we aimed to examine this association among older adults from six low- and middle-income countries (LMICs).

**Methods:**

Cross-sectional, nationally representative data from the Study on Global Ageing and Adult Health were analyzed. Data on 20,198 adults aged ≥60 years were analyzed [mean (SD) age 69.4 (13.1) years; 54.1% females]. Information on 11 chronic physical conditions was obtained. Dynapenia was defined as <26 kg for men and <16 kg for women. Abdominal obesity was defined as waist circumference of >88 cm for women and >102 cm for men. DAO was defined as having both dynapenia and abdominal obesity. Multivariable logistic regression was conducted.

**Results:**

After adjustment for potential confounders, compared to no dynapenia and no abdominal obesity, dynapenia alone, abdominal obesity alone, and DAO are associated with 1.34 (95% CI = 1.16–1.55), 1.64 (95% CI = 1.36–1.98), and 2.49 (95% CI = 1.94–3.19) times higher odds for physical multimorbidity, respectively.

**Conclusions:**

Dynapenic abdominal obesity is significantly associated with higher odds for physical multimorbidity among older adults in LMICs. Prevention and management of dynapenic abdominal obesity may aid in reducing the burden of physical multimorbidity, pending future longitudinal research.

## Plain language summary

We aimed to examine the association between low muscle strength and abdominal obesity with chronic medical conditions in older adults from 6 low- and middle-income countries (LMICs). We found that people with low muscle strength and abdominal obesity were more likely to have other chronic medical conditions. This finding highlights the need to investigate whether people with low muscle strength and abdominal obesity have other medical conditions, as this should allow them to be treated for these other conditions and hence have better overall health.

## Abstract (web summary)

Smith et al. examine the association between physical multimorbidity and dynapenic abdominal obesity in 20,198 adults aged ≥60 years from low- and middle-income countries. Dynapenic abdominal obesity is significantly associated with higher odds for physical multimorbidity.

## Introduction

Non-communicable diseases (NCDs) are responsible for ~71% of global deaths, and 77% of these deaths occur in low- and middle-income countries (LMICs)^1^. Moreover, NCDs are most common in older adults, and a substantial increase in NCD burden is expected due to rapid ageing, especially in the context of LMICs, where the United Nations estimates that two-thirds of the world’s population aged 60 years and over will live by 2050^2^.

When two or more physical chronic conditions exist simultaneously in an individual, this condition is called physical multimorbidity^3^. Multimorbidity is an important risk concept as it is associated with adverse health outcomes such as lower quality of life^4^, increased healthcare utilization^5^, and higher risk for premature mortality^6^. In the context of LMICs, this condition is particularly difficult to manage in many settings due to fragmented care and high costs for medical care. Given this background, it is important to identify risk factors for chronic physical conditions and physical multimorbidity among older adults in LMICs to aid in the development of targeted interventions.

In recent years, there has been increasing interest in dynapenic abdominal obesity (DAO) (i.e., impairment in muscle strength and high waist circumference)^7^ as it has been observed to be strongly associated with higher risk for adverse health outcomes such as falls^8^, metabolic alterations^8^, functional decline^9,10^, and premature mortality^11^. DAO may increase risk for physical chronic diseases and physical multimorbidity, as weak muscle strength has been found to be associated with an unfavorable inflammatory profile (e.g., higher levels of C-reactive proteins and fibrinogen)^12^, while the metabolic risk associated with obesity is closely correlated with a central rather than peripheral fat pattern^13^. Therefore, it is possible for people with DAO to be at particularly high risk for multimorbidity, but, as far as we are aware, there are currently no studies on this topic.

Therefore, the aim of the present study was to examine associations of DAO with chronic physical conditions and physical multimorbidity in a sample of 20,198 adults aged ≥60 years from six LMICs. Our main result is that DAO is significantly associated with higher odds for physical multimorbidity among older adults in LMICs.

## Methods

### The survey

We used data from the Study on Global Ageing and Adult Health (SAGE) Wave 1, conducted by the World Health Organization (WHO) between 2007 and 2010. We analyzed data from six countries: China, Ghana, India, Mexico, Russia, and South Africa. Access to the dataset was obtained by registering and submitting a request through the WHO SAGE website. Based on the World Bank classification at the time of the survey, Ghana was the only low-income country, and China and India were lower-middle-income countries, although China became an upper-middle-income country in 2010. The remaining countries were upper-middle-income countries. Details of the survey methodology have been published elsewhere^14^. Briefly, in order to obtain nationally representative samples, a multistage clustered sampling design method was used. The sample consisted of adults aged ≥18 years with oversampling of those aged ≥50 years. Trained interviewers conducted face-to-face interviews using a standard questionnaire. Standard translation procedures were undertaken to ensure comparability between countries. The survey response rates were: China 93%; Ghana 81%; India 68%; Mexico 53%; Russia 83%; and South Africa 75%. Sampling weights were constructed to adjust for the population structure as reported by the United Nations Statistical Division. Ethical approval was obtained from the WHO Ethical Review Committee and local ethics research review boards: the Ethics Review Committee of the Chinese Center for Disease Control and Prevention (China), the Ghana Health Service Ethical Review Committee (Ghana), the Indian Council of Medical Research Ethics Committee (India), the Comisión Nacional de Bioética (Mexico), the Ethics Committee of the National Research Center for Preventive Medicine, Russian Medical Academy of Postgraduate Education (Russia), and the Human Sciences Research Council Research Ethics Committee (South Africa). Written informed consent was obtained from all participants.

### Chronic physical conditions and physical multimorbidity

We included all 11 chronic physical conditions (angina, arthritis, asthma, chronic back pain, chronic lung disease, diabetes, edentulism, hearing problems, hypertension, stroke, visual impairment) for which data were available in the SAGE^11,15–20^. Chronic back pain was defined as having had back pain every day during the last 30 days. Respondents who answered affirmatively to the question “Have you lost all of your natural teeth?” were considered to have edentulism. The participant was considered to have hearing problems if the interviewer observed this condition during the survey. Hypertension was defined as having at least one of the following: systolic blood pressure ≥140 mmHg; diastolic blood pressure ≥90 mmHg; or self-reported diagnosis. Visual impairment was defined as having severe/extreme difficulty in seeing and recognizing a person that the participant knows across the road^21^. Diabetes and stroke were solely based on lifetime self-reported diagnosis. For other conditions, the participant was considered to have the condition in the presence of either one of the following: self-reported diagnosis, or symptom-based diagnosis based on algorithms. We used these algorithms, which have been used in previous studies using the same dataset, to detect undiagnosed cases^22,23^. Specifically, the validated Rose questionnaire was used for angina^24^, and other previously validated symptom-based algorithms were used for arthritis, asthma, and chronic lung disease^22^. Further details on the definition of chronic physical conditions can be found in Supplementary Data 2. Multimorbidity was defined as ≥2 chronic physical conditions, in line with previously used definitions^23^.

### Dynapenia, abdominal obesity, and dynapenic abdominal obesity

Handgrip strength was measured using a Smedley Hand Dynamometer (Scandidact Aps, Denmark). Dynapenia was defined as <26 kg for men and <16 kg for women^25^, using the average value of the two handgrip measurements of the dominant hand. Waist circumference was measured at the midpoint between the lower margin of the least palpable rib and the top of the iliac crest, keeping the measuring tape parallel to the floor. Abdominal obesity was defined as a waist circumference of >88 cm for women and >102 cm for men^26^. Participants were divided into four groups according to dynapenia and abdominal obesity status: No dynapenia and no abdominal obesity, dynapenia alone, abdominal obesity alone, and dynapenia and abdominal obesity (DAO).

### Control variables

The selection of the control variables was based on past literature^27^ and included age, sex, highest level of education achieved (≤primary, secondary, tertiary), country-wise wealth quintiles based on income, physical activity, alcohol consumption, and smoking (never, current, past). Levels of physical activity were assessed with the Global Physical Activity Questionnaire and were classified as low, moderate, and high based on conventional cut-offs^28^. Consumers of at least four (females) or five drinks (males) of any alcoholic beverage per day on at least 1 day in the past week were considered “heavy” drinkers. Those who had ever consumed alcohol but were not heavy drinkers were categorized as “non-heavy” drinkers^29^.

### Statistical analysis

The statistical analysis was performed with Stata 14.2 (Stata Corp LP, College Station, Texas). The analysis was restricted to those aged ≥60 years. The difference in sample characteristics between those with and without physical multimorbidity was tested by Chi-squared tests, except for age (Student’s *t*-test). Multivariable logistic regression analysis^30^ was performed to examine the association between the four-category variables of dynapenia, abdominal obesity, or DAO (exposure) and the individual chronic conditions or physical multimorbidity (outcomes), with no dynapenia and no abdominal obesity being the reference category. Sex-stratified analyses were conducted for the analysis with physical multimorbidity as the outcome. The regression analyses were adjusted for age, sex, education, wealth, physical activity, alcohol consumption, smoking, and country, with the exception of the sex-stratified analysis, which was not adjusted for sex. Adjustment for country was done by including dummy variables for each country in the model, as in previous SAGE publications^31,32^. The sample weighting and the complex study design were taken into account in the analyses. Results from the regression analyses are presented as odds ratios (ORs) with 95% confidence intervals (CIs). The level of statistical significance was set at P < 0.05.

### Reporting summary

Further information on research design is available in the Nature Portfolio Reporting Summary linked to this article.

## Results

**Fig. 1:**
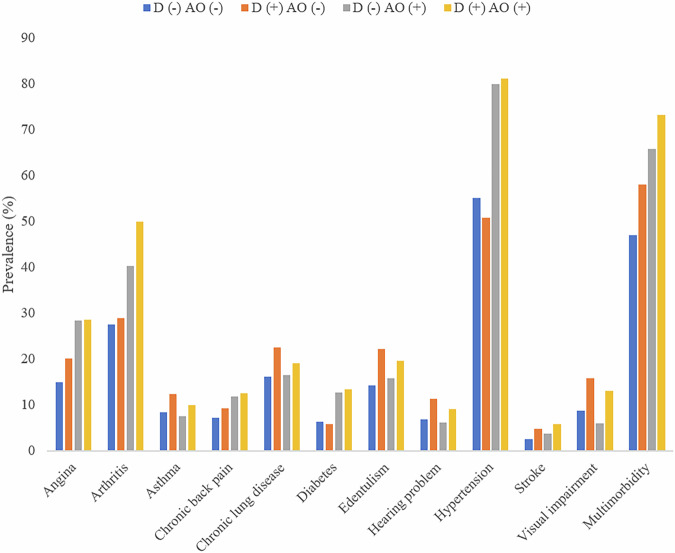
Prevalence of chronic disease or physical multimorbidity by dynapenia, abdominal obesity, or both. D dynapenia, AO abdominal obesity. Physical multimorbidity was defined as ≥2 chronic physical conditions.

Data on 20,198 adults aged ≥60 years were analyzed. The sample sizes of each country were: China n = 7474; Ghana n = 2616; India n = 3621; Mexico n = 1879; Russia n = 2465; South Africa n = 2143. The prevalence of dynapenia alone, abdominal obesity alone, and DAO were 33.3%, 15.8%, and 7.7%, respectively, while the prevalence of physical multimorbidity was 57.3%. The sample characteristics are provided in Table 1. The mean (SD) age was 69.4 (13.1) years, and 54.1% were females. People with physical multimorbidity were significantly more likely to be older, female, and with lower levels of wealth. Furthermore, they were more likely to be past smokers, have lower levels of physical activity, and less likely to be heavy drinkers. The most prevalent disease pair among people with DAO was hypertension and arthritis (41.3%), followed by hypertension and angina (24.8%) (Table S1). The prevalence of physical multimorbidity among those without dynapenia nor abdominal obesity, dynapenia alone, abdominal obesity alone, and DAO was 47.1%, 58.0%, 65.9%, and 73.3%, respectively (Fig. 1). After adjustment for potential confounders, compared to no dynapenia and no abdominal obesity, DAO was significantly associated with higher odds for angina, arthritis, asthma, chronic back pain, diabetes, hypertension, and stroke (OR = 1.56–2.95) (Table 2). Furthermore, dynapenia alone, abdominal obesity alone, and DAO were associated with 1.34 (95% CI = 1.16–1.55), 1.64 (95% CI = 1.36–1.98), and 2.49 (95% CI = 1.94–3.19) times higher odds for physical multimorbidity in the overall sample, respectively (Table 3). The OR of DAO was particularly high among males (OR = 4.61; 95% CI = 2.60–8.17) compared to females (OR = 2.33; 95% CI = 1.77–3.07).

**Table 1:** Sample characteristics (overall and by physical multimorbidity)

			Physical multimorbidity		
Characteristic		Overall	No	Yes	P-value^a^
Age	Mean (SD)	69.4 (13.1)	67.6 (12.0)	70.5 (13.4)	<0.001
Sex	Female	54.1	46.9	59.5	<0.001
	Male	45.9	53.1	40.5	
Education	≤Primary	63.9	66.0	62.1	0.051
	Secondary	29.8	27.3	31.8	
	Tertiary	6.3	6.7	6.1	
Wealth	Poorest	20.1	18.8	21.1	0.008
	Poorer	20.2	18.8	21.3	
	Middle	20.5	20.0	20.8	
	Richer	18.8	19.4	18.2	
	Richest	20.3	23.0	18.6	
Smoking	Never	60.6	57.6	62.4	<0.001
	Smoker	31.6	35.8	28.9	
	Past	7.8	6.5	8.7	
Physical activity	High	40.0	45.3	36.5	<0.001
	Moderate	24.8	25.2	24.7	
	Low	35.2	29.4	38.9	
Alcohol consumption	Never	67.8	69.8	66.4	0.011
	Non-heavy	29.2	26.6	31.1	
	Heavy	3.0	3.5	2.6	

Data are % unless otherwise stated.

*SD* standard deviation.

^a^P-value was based on Chi-squared tests except for age (Student’s *t*-test).

**Table 2:** Assocation between dynapenia, abdominal obesity, or both, and individual physical conditions (outcome) estimated by multivariable logistic regression

	D ( + ) AO (−)		D (−) AO (+)		D ( + ) AO (+)	
Outcome	OR	95% CI	OR	95% CI	OR	95% CI
Angina	1.44***	[1.20,1.73]	1.32*	[1.05,1.65]	1.87***	[1.40,2.49]
Arthritis	1.03	[0.88,1.20]	1.49***	[1.24,1.78]	2.28***	[1.82,2.85]
Asthma	1.21	[0.92,1.58]	1.34	[0.93,1.92]	1.69*	[1.09,2.63]
Chronic back pain	1.17	[0.86,1.60]	1.35	[0.98,1.86]	1.56*	[1.05,2.33]
Chronic lung disease	1.44***	[1.18,1.75]	0.90	[0.70,1.15]	1.30	[0.97,1.76]
Diabetes	0.90	[0.69,1.17]	2.23***	[1.70,2.92]	2.16***	[1.57,2.96]
Edentulism	1.22*	[1.01,1.48]	0.92	[0.73,1.14]	1.00	[0.72,1.39]
Hearing problem	1.29*	[1.04,1.60]	0.73	[0.54,1.00]	0.94	[0.64,1.38]
Hypertension	0.98	[0.84,1.15]	2.39***	[1.84,3.10]	2.95***	[2.12,4.10]
Stroke	2.06***	[1.53,2.79]	1.21	[0.75,1.97]	1.90**	[1.23,2.94]
Visual impairment	1.23	[0.95,1.59]	0.72	[0.51,1.02]	1.37	[0.93,2.03]

Reference category is dynapenia (−) abdominal obesity (−).

Models are adjusted for age, sex, education, wealth, physical activity, alcohol consumption, smoking, and country.

Physical multimorbidity was defined as ≥2 chronic physical conditions.

*OR* odds ratio, *CI* confidence interval, *D* dynapenia, *AO* abdominal obesity.

*p < 0.05, **p < 0.01, ***p < 0.001.

**Table 3:** Assocation between dynapenia, abdominal obesity, or both, and physical multimorbidity (outcome) estimated by multivariable logistic regression

	Overall		Male		Female	
Exposure	OR	95% CI	OR	95% CI	OR	95% CI
D (−) AO (−)	1.00		1.00		1.00	
D (+) AO (−)	1.34***	[1.16,1.55]	1.34***	[1.14,1.57]	1.34*	[1.04,1.71]
D (−) AO (+)	1.64***	[1.36,1.98]	2.15**	[1.30,3.55]	1.58***	[1.28,1.97]
D (+) AO (+)	2.49***	[1.94,3.19]	4.61***	[2.60,8.17]	2.33***	[1.77,3.07]

Models are adjusted for age, sex, education, wealth, physical activity, alcohol consumption, smoking, and country with the exception of the sex-stratified analysis, which was not adjusted for sex.

Physical multimorbidity was defined as ≥2 chronic physical conditions.

*OR* odds ratio; *CI* confidence interval; *D* dynapenia; *AO* abdominal obesity.

*p < 0.05, **p < 0.01, ***p < 0.001.

## Discussion

In the present study, including large representative samples of older adults from six LMICs, compared to no dynapenia and no abdominal obesity, DAO was significantly associated with higher odds for angina, arthritis, asthma, chronic back pain, diabetes, hypertension, and stroke. Moreover, DAO was associated with 2.49 (95% CI = 1.94–3.19) times higher odds for physical multimorbidity in the overall sample. This association was particularly pronounced among males (OR = 4.61; 95% CI = 2.60–8.17). To the best of our knowledge, this is the first study on DAO and physical multimorbidity.

DAO is characterized by low muscle strength and a high level of central adiposity. Indeed, low muscle strength and central adiposity are both associated with an unfavorable inflammatory profile. Animal studies have shown that administration of interleukin (IL)−6 or tumor necrosis factor (TNF)-α increases skeletal muscle breakdown, decreases the rate of protein synthesis, and inhibits plasma concentrations of insulin-like growth factor that may impair muscle anabolic processes^33–35^. In humans, cross-sectional studies have shown associations between various inflammatory markers and objective markers of muscle strength, as assessed by handgrip and knee extensor measures^36,37^. Importantly, central adiposity involves impairment of immune response affecting both innate and adaptive immunity, and adipose tissue can produce and secrete inflammatory molecules such as tumor necrosis factor-α (TNF-α) and interleukin-1 receptor-associated kinase (IRAK)−1^38^. Dysregulation of inflammatory response contributes broadly to the development of many chronic conditions. Indeed, in chronic inflammation, immune cells become dysregulated and lose self-limiting nature^39^. Thus, inflammation is a key mechanism that likely explains the relationship between DAO and several chronic conditions as well as physical multimorbidity.

When considering individual chronic conditions, DAO is likely associated with angina, hypertension, and stroke owing to inflammation. However, DAO is also associated with lower plasma HDL-cholesterol and higher triglycerides^40^ both of which have been implicated in the development of all these conditions^41^. DAO is likely associated with an unfavorable cholesterol profile owing to a loss of muscle strength, thus skeletal muscle per se, impacting regulators of carbohydrate and lipid metabolism. Reduced skeletal muscle mass can severely weaken the function of the musculoskeletal system and impair lipid homeostasis, particularly in the obese state. Moreover, central adiposity is associated with a cluster of lipid abnormalities, such as hypercholesterolemia, high fasting triglyceride levels, low high-density lipoprotein cholesterol, and high apolipoprotein B^42^. Interestingly, Table 3 demonstrates that in the present study, abdominal obesity is the predominant driver of the association between DAO and hypertension and diabetes. An increase in both visceral abdominal adipose tissue and intermuscular fat, markers of DAO, is associated with insulin resistance, and this likely explains the higher risk for diabetes among those with DAO^11,43^.

Next, a higher risk of arthritis in DAO may be mainly explained by central adiposity. Specifically, this may be due to the biomechanical effect of being overweight and the metabolic or inflammatory effects related to central obesity^44^. Finally, in terms of asthma, DAO can potentially increase risk for asthma via chronic inflammation, as previously discussed^11,45^. The accumulation of the above mechanisms, resulting in multiple chronic conditions, may explain the strong association between DAO and multimorbidity observed in our study.

The finding that the association between DAO and multimorbidity was more pronounced in males than in females is interesting and should be noted. Similarly, in a study including 6183 individuals aged ≥60 years from the English Longitudinal Study of Ageing, it was found that DAO was associated with a stronger decline in physical performance in males but not in females^46^. These findings are likely owing to the differing patterns of fat accumulation between the sexes. Indeed, males exhibit more age-related loss of muscle strength and accumulate abdominal fat earlier, with greater intensity, and with a predisposition toward visceral fat deposition^46^. Importantly, visceral fat deposition is strongly associated with multiple chronic conditions, including, for example, cardiometabolic diseases^47^ and mild cognitive impairment^48^, and this is likely owing to visceral fat being associated with higher insulin resistance, inflammation, and small vessel disease^48^. Moreover, previous literature has demonstrated that DAO was significantly associated with activities of daily living (ADL) disability only in men^49^, while ADL *per se* has been found to be associated with chronic conditions^50^ and self-rated health^51^. However, further research of an experimental nature is now needed to identify the predominant mechanisms that drive the stronger observed association between DAO and multimorbidity in males compared to females.

Findings from this present study show that DAO is associated with higher odds for several chronic physical diseases and physical multimorbidity. If findings are confirmed by longitudinal studies, interventions to improve or maintain muscle strength and reduce excess body fat may have an important role in decreasing chronic disease burden among older adults. Such interventions could focus on promotion of physical activity, strength training, and nutrition as these have been shown to be successful in relation to sarcopenia management and prevention^52–55^. Furthermore, approaches intended to promote obesity prevention in the entire population include subsidizing nutritious food, or conversely, taxing unhealthy foods^56^. In addition, although not restricted to obese individuals, the Exercise is Medicine Global Initiative is designed to support healthcare professionals in prescribing exercise for patients by training providers to assess patient physical activity levels, imparting behavioral counseling to increase activity using change models, and referring patients to resources to facilitate physical activity. Indeed, this initiative already exists in LMICs^56^. Finally, screening for DAO in both community and clinical settings may help identify those who are at particularly high risk of developing chronic physical diseases and physical multimorbidity.

The large representative samples of older adults across multiple LMICs are clear strengths of the present study. However, the present findings must be interpreted in light of the study limitations. First, the study is cross-sectional in nature, and thus, the direction of the association cannot be determined. In fact, it is possible for DAO to cause physical diseases, and also for physical diseases to cause DAO via poorer diets or limitations in mobility leading to highly sedentary lifestyles, and this can create a vicious cycle where one condition exacerbates the other. Second, the majority of variables were self-reported, potentially introducing recall and social desirability bias into the findings. For example, some conditions were only based on self-reported diagnosis (e.g., diabetes). For other conditions, we attempted to reduce underdiagnosis by the use of an algorithm based on symptoms, but the use of non-validated instruments for some conditions may have led to higher sensitivity at the expense of lower specificity. Third, although the list of chronic conditions in our study included most diseases which are highly prevalent in old age, it lacked diseases or conditions such as cancer, hypercholesterolemia, heart failure, and chronic kidney disease, which have been linked with obesity^57–59^. Thus, it is possible for the results to have changed with a different list of chronic conditions. Finally, the present dataset did not contain information on participant medication, and thus, it is possible that this has introduced some level of residual confounding into the analysis. For example, some drugs are known to cause weight gain.

## Conclusion

In the present study, including large representative samples of older adults across six LMICs, DAO was significantly associated with higher odds for multiple individual chronic conditions and physical multimorbidity. Future longitudinal studies are needed to assess the impact of improvements or maintenance of muscle strength and reduction in excess body fat on chronic conditions and physical multimorbidity among older people. The present findings, taken together with the broader literature, suggest that it may be prudent to screen for DAO in both community and clinical settings to help identify those who are at particularly high risk of developing chronic physical diseases and physical multimorbidity.

## Supplementary information

Supplementary Information

Description of Additional Supplementary Files

Supplementary Data 1

Supplementary Data 2

Reporting Summary

## Data Availability

The data used in this study are publicly available from the World Health Organization’s Study on Global Ageing and Adult Health (SAGE). Researchers can request access to the datasets by registering at the WHO SAGE data portal: https://www.who.int/data/data-collection-tools/study-on-global-ageing-and-adult-health. The source data for Fig. 1 are in Supplementary Data 1.

## References

[1] World Health Organization. Noncommunicable diseases. Accessed 16 March 2025, Available at: https://www.who.int/news-room/fact-sheets/detail/noncommunicable-diseases (2021).

[2] United Nations. *World Population Ageing 2019 Highlights* (United Nations, 2019).

[3] NICE. The National Institute for Health and Care Excellence. Multimorbidity: clinical assessment and management. Accessed 16 March 2025, Available at: https://www.nice.org.uk/guidance/ng56/chapter/recommendations#multimorbidity (2016).

[4] Fortin, M. et al. Multimorbidity and quality of life in primary care: a systematic review. *Health Qual. Life Outcomes* **2**, 1–12 (2004). 15380021 10.1186/1477-7525-2-51 PMC526383

[5] Lehnert, T. et al. Health care utilization and costs of elderly persons with multiple chronic conditions. *Med. Care Res. Rev.* **68**, 387–420 (2011). 21813576 10.1177/1077558711399580

[6] Gallo, J. J. et al. Multimorbidity, depression, and mortality in primary care: randomized clinical trial of an evidence-based depression care management program on mortality risk. *J. Gen. Intern. Med.* **31**, 380–386 (2016). 26432693 10.1007/s11606-015-3524-y PMC4803701

[7] de Oliveira Máximo, R. et al. Dynapenia, abdominal obesity or both: which accelerates the gait speed decline most?. *Age Ageing* **50**, 1616–1625 (2021). 34087934 10.1093/ageing/afab093 PMC8437070

[8] de Oliveira Máximo, R. et al. Abdominal obesity, dynapenia and dynapenic-abdominal obesity as factors associated with falls. *Braz. J. Phys. Ther.* **23**, 497–505 (2019). 30391361 10.1016/j.bjpt.2018.10.009 PMC6849078

[9] Alexandre, T., da, S., Scholes, S., Santos, J. L. F. & De Oliveira, C. Dynapenic abdominal obesity as a risk factor for worse trajectories of ADL disability among older adults: the ELSA cohort study. *J. Gerontol. Ser. A* **74**, 1112–1118 (2019). 10.1093/gerona/gly182 PMC6580691 30165562

[10] Kushkestani, M., Parvani, M., Ghafari, M. & Avazpoor, Z. The role of exercise and physical activity on aging-related diseases and geriatric syndromes. *SPORT TK Rev. EuroAm. Cienc. Deporte* **11**, 6 (2022).

[11] Rossi, A. P. et al. Dynapenic abdominal obesity as predictor of mortality and disability worsening in older adults: a 10-year prospective study. *Clin. Nutr.* **35**, 199–204 (2016). 25736030 10.1016/j.clnu.2015.02.005

[12] Smith, L., Yang, L. & Hamer, M. Handgrip strength, inflammatory markers, and mortality. *Scand. J. Med. Sci. Sports* **29**, 1190–1196 (2019). 30972827 10.1111/sms.13433

[13] Shen, W. et al. Waist circumference correlates with metabolic syndrome indicators better than percentage fat. *Obesity* **14**, 727–736 (2006). 16741276 10.1038/oby.2006.83 PMC1894647

[14] Kowal, P. et al. Data resource profile: the World Health Organization Study on global AGEing and adult health (SAGE). *Int. J. Epidemiol.* **41**, 1639–1649 (2012). 23283715 10.1093/ije/dys210 PMC3535754

[15] Wang, X. et al. Dynapenic abdominal obesity and risk of heart disease among middle-aged and older adults: a prospective cohort study. *J. Nutr. Heal. Aging* **27**, 752–758 (2023). 10.1007/s12603-023-1975-0 PMC12275555 37754215

[16] Zhou, S. et al. Association between dynapenic abdominal obesity and arthritis among the middle-aged and older Chinese: a longitudinal study. *Aging Clin. Exp. Res.* **36**, 198 (2024). 39367987 10.1007/s40520-024-02847-y PMC11455664

[17] Jiang, D., Wang, L., Bai, C. & Chen, O. Association between abdominal obesity and asthma: a meta-analysis. *Allergy Asthma Clin. Immunol.* **15**, 1–11 (2019). 30949213 10.1186/s13223-019-0333-6 PMC6431003

[18] Naumov, A. V. et al. Chronic pain in patients older than 60 years: a view of the geriatrics. Zh. Nevrol. Psikhiatr. Im. SS Korsakova **119**, 53–59 (2019). 10.17116/jnevro201911906153 31407682

[19] Cuthbertson, D. J. et al. Dynapenic obesity and the risk of incident Type 2 diabetes: the English longitudinal study of ageing. *Diabet. Med.* **33**, 1052–1059 (2016). 26479063 10.1111/dme.12991

[20] Yamaguchi, K. et al. Factors associated with masseter muscle quality assessed from ultrasonography in community-dwelling elderly individuals: a cross-sectional study. *Arch. Gerontol. Geriatr.* **82**, 128–132 (2019). 30780049 10.1016/j.archger.2019.02.003

[21] Freeman, E. E. et al. The global burden of visual difficulty in low, middle, and high income countries. *PLoS ONE* **8**, e63315 (2013). 23675477 10.1371/journal.pone.0063315 PMC3651198

[22] Arokiasamy, P. et al. Chronic noncommunicable diseases in 6 low-and middle-income countries: findings from wave 1 of the World Health Organization’s study on global Ageing and adult health (SAGE). *Am. J. Epidemiol.* **185**, 414–428 (2017). 28399566 10.1093/aje/kww125 PMC6075549

[23] Garin, N. et al. Global multimorbidity patterns: a cross-sectional, population-based, multi-country study. *J. Gerontol. Ser. A Biomed. Sci. Med. Sci.* **71**, 205–214 (2016). 10.1093/gerona/glv128 PMC5864156 26419978

[24] Rose, G. A. The diagnosis of ischaemic heart pain and intermittent claudication in field surveys. *Bull. World Health Organ.* **27**, 645 (1962). 13974778 PMC2555832

[25] Studenski, S. A. et al. The FNIH sarcopenia project: rationale, study description, conference recommendations, and final estimates. *J. Gerontol. A Biol. Sci. Med. Sci.* **69**, 547–558 (2014). 24737557 10.1093/gerona/glu010 PMC3991146

[26] World Health Organization. *Waist Circumference and Waist-Hip Ratio: Report of a WHO Expert Consultation, Geneva, 8-11 December 2008* (World Health Organization, 2011).

[27] An, K. O. & Kim, J. Association of sarcopenia and obesity with multimorbidity in Korean adults: a nationwide cross-sectional study. *J. Am. Med. Dir. Assoc.* **17**, 960.e1–e7 (2016). 10.1016/j.jamda.2016.07.005 27567461

[28] Bull, F. C., Maslin, T. S. & Armstrong, T. Global physical activity questionnaire (GPAQ): nine country reliability and validity study. *J. Phys. Act. Health* **6**, 790–804 (2009). 20101923 10.1123/jpah.6.6.790

[29] Koyanagi, A. et al. The association between obesity and back pain in nine countries: a cross-sectional study. *BMC Public Health* **15**, 1–9 (2015). 25886589 10.1186/s12889-015-1362-9 PMC4331391

[30] Qi, C. & Hu, L. Exploration of innovative learning ability cultivation based on logistic regression. *Appl. Math. Nonlinear Sci.* **7**, 1085–1092 (2022).

[31] Koyanagi, A. et al. Chronic physical conditions, multimorbidity, and mild cognitive impairment in low-and middle-income countries. *J. Am. Geriatr. Soc.* **66**, 721–727 (2018). 29427504 10.1111/jgs.15288 PMC5906176

[32] Koyanagi, A. et al. Chronic conditions and sleep problems among adults aged 50 years or over in nine countries: a multi-country study. *PLoS ONE* **9**, e114742 (2014). 25478876 10.1371/journal.pone.0114742 PMC4257709

[33] Charters, Y. & Grimble, R. F. Effect of recombinant human tumour necrosis factor α on protein synthesis in liver, skeletal muscle and skin of rats. *Biochem. J.* **258**, 493–497 (1989). 2468333 10.1042/bj2580493 PMC1138388

[34] García-Martínez, C., López-Soriano, F. J. & Argilés, J. M. Acute treatment with tumour necrosis factor-α induces changes in protein metabolism in rat skeletal muscle. *Mol. Cell. Biochem.* **125**, 11–18 (1993). 8264567 10.1007/BF00926829

[35] De Benedetti, F. et al. Interleukin 6 causes growth impairment in transgenic mice through a decrease in insulin-like growth factor-I. A model for stunted growth in children with chronic inflammation. *J. Clin. Investig.* **99**, 643–650 (1997). 9045866 10.1172/JCI119207 PMC507846

[36] Taaffe, D. R., Harris, T. B., Ferrucci, L., Rowe, J. & Seeman, T. E. Cross-sectional and prospective relationships of interleukin-6 and C-reactive protein with physical performance in elderly persons: MacArthur studies of successful aging. *J. Gerontol. Ser. A Biol. Sci. Med. Sci.* **55**, M709–M715 (2000). 11129392 10.1093/gerona/55.12.m709

[37] Visser, M. et al. Relationship of interleukin-6 and tumor necrosis factor-α with muscle mass and muscle strength in elderly men and women: the Health ABC Study. *J. Gerontol. Ser. A Biol. Sci. Med. Sci.* **57**, M326–M332 (2002). 11983728 10.1093/gerona/57.5.m326

[38] Liu, D. et al. The association between normal BMI with central adiposity and proinflammatory potential immunoglobulin GN-glycosylation. *Diabetes Metab. Syndr. Obes. Targets Ther.* **12**, 2373–2385 (2019). 10.2147/DMSO.S216318 PMC6861528 31814749

[39] Zhong, J. & Shi, G. Regulation of inflammation in chronic disease. *Front. Immunol.* **10**, 737 (2019). 31031750 10.3389/fimmu.2019.00737 PMC6473051

[40] Sénéchal, M., Dionne, I. J. & Brochu, M. Dynapenic abdominal obesity and metabolic risk factors in adults 50 years of age and older. *J. Aging Health* **24**, 812–826 (2012). 22451528 10.1177/0898264312440324

[41] Pearson, T. A., Bulkley, B. H., Achuff, S. C., Kwiterovich, P. O. & Gordis, L. The association of low levels of HDL cholesterol and arteriographically defined coronary artery disease. *Am. J. Epidemiol.* **109**, 285–295 (1979). 222134 10.1093/oxfordjournals.aje.a112682

[42] Gong, H., Liu, Y., Lyu, X., Dong, L. & Zhang, X. Lipoprotein subfractions in patients with sarcopenia and their relevance to skeletal muscle mass and function. *Exp. Gerontol.* **159**, 111668 (2022). 34954281 10.1016/j.exger.2021.111668

[43] López Sánchez, G. F. et al. Impact of physical activity, BMI and sociodemographic and lifestyle factors on the risk of diabetes in 9511 Ghanaian adults. *SPORT TK Rev. EuroAm. Cienc. Deporte* **11**, 15 (2022).

[44] Tavares, N. H. C. et al. How does global and central obesity influences on arthritis or rheumatism? Results from the Brazilian National Health Survey, 2013. *Int. J. Clin. Pract.* **75**, e14027 (2021). 34233399 10.1111/ijcp.14027

[45] Murdoch, J. R. & Lloyd, C. M. Chronic inflammation and asthma. *Mutat. Res. Mol. Mech. Mutagen.* **690**, 24–39 (2010). 10.1016/j.mrfmmm.2009.09.005 PMC2923754 19769993

[46] Máximo, R. et al. Combination of dynapenia and abdominal obesity affects long-term physical performance trajectories in older adults: sex differences. *Am. J. Clin. Nutr.* **115**, 1290–1299 (2022). 35102379 10.1093/ajcn/nqac023 PMC9071386

[47] He, Q. et al. Visceral adiposity associated with incidence and development trajectory of cardiometabolic diseases: a prospective cohort study. *Nutr. Metab. Cardiovasc. Dis.* **34**, 1235–1244 (2024). 38331642 10.1016/j.numecd.2023.12.024

[48] Chiba, I. et al. Visceral fat accumulation is associated with mild cognitive impairment in community-dwelling older Japanese women. *J. Nutr. Health Aging***24**, 352–357 (2020).32115619 10.1007/s12603-020-1330-7

[49] Smith, L. et al. Sex differences in the association between dynapenic abdominal obesity and onset of disability in activities of daily living among adults aged ≥ 50 years: a prospective analysis of the Irish Longitudinal Study on Ageing. *Maturitas* **176**, 107763 (2023). 37393660 10.1016/j.maturitas.2023.04.006

[50] Han, W. et al. Trajectories of activities of daily living/instrumental activities of daily living and the risk of cardiovascular diseases. *Nutr. Metab. Cardiovasc. Dis.* **35**, 103969 (2025). 40180828 10.1016/j.numecd.2025.103969

[51] Gama, E. V. et al. Association of individual activities of daily living with self-rated health in older people. *Age Ageing* **29**, 267–270 (2000). 10855912 10.1093/ageing/29.3.267

[52] Cruz-Jentoft, A. J. et al. Prevalence of and interventions for sarcopenia in ageing adults: a systematic review. Report of the International Sarcopenia Initiative (EWGSOP and IWGS). *Age Ageing* **43**, 748–759 (2014). 25241753 10.1093/ageing/afu115 PMC4204661

[53] Morley, J. E. et al. Nutritional recommendations for the management of sarcopenia. *J. Am. Med. Dir. Assoc.* **11**, 391–396 (2010). 20627179 10.1016/j.jamda.2010.04.014 PMC4623318

[54] Sánchez García, C., Zauder, R. & López Sánchez, G. F. Analysis of body composition and physical fitness of futsal players at school age according to their level of physical activity, diet and body image. *Atena J. Sport. Sci.* **1**, 4 (2019).

[55] Rodríguez Cabeo, D. & Inglés López, M. The relationship between body image and muscle strength in Spanish children and adolescents. *Atena J. Public Heal.* **2**, 4 (2020).

[56] Ford, N. D., Patel, S. A. & Narayan, K. M. V. Obesity in low-and middle-income countries: burden, drivers, and emerging challenges. *Annu. Rev. Public Health* **38**, 145–164 (2017). 28068485 10.1146/annurev-publhealth-031816-044604

[57] Centers for Disease Control and Prevention. Obesity and Cancer. Accessed 16 March 2025, Available at: https://www.cdc.gov/cancer/obesity/index.htm (2022).

[58] Yang, C. et al. Interaction of general obesity and abdominal obesity with frailty in patients with chronic kidney disease: a nationally representative analysis. *Clin. Kidney J.* **17**, sfae142 (2024). 38983651 10.1093/ckj/sfae142 PMC11231580

[59] Ahmed, A., Blackman, M. R., White, M. & Anker, S. D. Emphasis on abdominal obesity as a modifier of eplerenone effect in heart failure: hypothesis-generating signals from EMPHASIS-HF. *Eur. J. Heart Fail.* **19**, 1198 (2017). 28560824 10.1002/ejhf.884 PMC7887704

