# Supplementary material for: Physical multimorbidity and dynapenic abdominal obesity among older adults from low- and middle-income countries
Source: Commun Med (Lond). 2025 Jul 29;5:315. doi: 10.1038/s43856-025-01037-9 (PMC12307868; doi:10.1038/s43856-025-01037-9)
Supplement: Supplementary file 1 — Supplementary Information [file 43856_2025_1037_MOESM1_ESM.pdf]

Table S1 Prevalence of each pair of chronic physical condition among people with dynapenic abdominal obesity

	Angina	Arthritis	Asthma	Chronic back pain	Chronic lung disease	Diabetes	Edentu-lism	Hearing problems	Hyperte-nsion	Stroke
Arthritis	17.8%									
Asthma	5.3%	6.6%								
Chronic back pain	5.6%	9.5%	2.0%							
Chronic lung disease	10.7%	11.4%	7.3%	4.8%						
Diabetes	4.2%	7.5%	1.5%	2.1%	2.7%					
Edentulism	5.9%	11.3%	1.4%	3.6%	4.8%	2.6%				
Hearing problems	2.7%	5.3%	1.8%	1.3%	2.4%	1.5%	3.0%			
Hypertension	24.8%	41.3%	8.3%	10.0%	15.9%	11.8%	17.2%	7.5%		
Stroke	2.4%	3.8%	0.5%	0.8%	1.1%	1.7%	1.4%	0.7%	5.6%	
Visual impairment	5.4%	6.9%	2.1%	2.7%	4.2%	2.4%	4.4%	3.0%	9.2%	0.7%

0%	10%	20%	30%	40%
